# Parent training for the treatment of irritability in children and adolescents: a multisite randomized controlled, 3-parallel-group, evaluator-blinded, superiority trial

**DOI:** 10.1186/s40359-022-00984-5

**Published:** 2022-11-22

**Authors:** E. Fongaro, M. C. Picot, A. Stringaris, C. Belloc, A. S. Verissimo, N. Franc, D. Purper-Ouakil

**Affiliations:** 1 Centre Hospitalo-Universitaire de Montpellier, Service Médecine Psychologique de l’Enfant et de l’Adolescent, Montpellier, Hérault France; 2 INSERM U 1018, CESP, Developmental Psychiatry/ADHD and Emotional Disorders, Villejuif, Île-de-France France; 3 Centre Hospitalo-Universitaire de Montpellier, Unité de Recherche Clinique and Epidémiologie, DIM, Montpellier, Hérault France; 4 Division of Psychiatry, Department of Clinical, Educational and Health Psychology, University College London, London, UK; 5 Department of Psychiatry, National and Kapodistrian University of Athens, Athens, Greece; 6 Centre Hospitalo-Universitaire de Montpellier, Direction de la Recherche et à l’Innovation (DRI), Montpellier, Hérault France

**Keywords:** Irritability, ADHD, Parental coping strategies, Developmental disorders, Non-violent resistance, Parent management training

## Abstract

**Background:**

Irritability is common in children and adolescents with Attention Deficit Hyperactivity Disorder (ADHD), Oppositional Defiant Disorder (ODD) and with anxiety/depressive disorders. Although youth irritability is linked with psychiatric morbidity, little is known regarding its non-pharmacological treatments. Developing non-pharmacological treatments for children with severe, chronic irritability is an important target for clinical research. To achieve this goal, we will test the benefits of parent-focused therapies in reducing irritability. The aim of the study is to compare Parent Management Training (PMT) and Non-Violent Resistance Training (NVR) programs with treatment-as-usual (TAU) on the improvement of irritability in children and adolescents with a baseline Parent-rated Affective Reactivity Index of 4 or higher, in the context of ADHD and other emotional and behavioural disorders. Additionally, we will assess (i) improvement of irritability at different times and according to different informants (parents, children, clinicians); (ii) improvement of parental strategies; and (iii) acceptability of the interventions, exploring possible mechanisms of the therapeutic effect.

**Methods:**

Two hundred and seventy participants between 6 and 15 years with ADHD and other emotional and behavioural disorders will be recruited and randomly assigned with their parents to the PMT, NVR, and TAU groups. PMT and NVR programs have 10 online sessions and two booster sessions at 1 and at 3 months. The primary outcome measure is the change from baseline at 3 months after completion of the program of the Clinician-rated Affective Rating Scale (CL-ARI) assessed by a blind evaluator. Secondary outcome measures include the change from baseline from those scales: the CL-ARI, the Clinical Global Impression Improvement scale, the Parenting and Familial Adjustment Scales, the Child-rated Cranky thermometers and the Parent-rated ARI. We will assess the parent’s expressed emotions and reflexivity during the online five-minute speech sample, clinical dimensions through the Child Behavior Checklist 6–18 and the Inventory of Callous Unemotional traits. Evaluations will be done remotely at baseline and at 1- and 3-months follow-up visits.

**Discussion:**

We expect a benefit in controlling irritability in the treatment groups. This will constitute an important achievement in promoting parental support programs in the treatment of irritability in the context of emotional and behavioural disorders.

Clinicaltrials.gov. Number: NCT05528926. Registered on the 2nd of September, 2022.

## Background

### Background and rationale {6a}

Irritability is defined as developmentally inappropriate proneness to anger [1]. Irritability is a symptom of several mental health conditions in children and adolescents such as attention deficit hyperactivity disorder (ADHD), oppositional defiant disorder (ODD), conduct disorder (CD), depressive disorders, and anxiety disorders. Both tonic (e.g., negative quality of mood) and phasic (e.g., temper outbursts) irritability are criteria of the Disruptive Mood Dysregulation Disorder reported in the Diagnostic and Statistical Manual of Mental Disorders (DSM–5) [2, 3].

Irritability is associated with poor functional outcomes across the lifespan [4, 5], with concurrent and longitudinal emotional disorders [6, 7], suicidality [8], and impaired academic and socio-professional functioning [5]. Children with high irritability also have distinct physiological profiles with hyper-reactivity to stress and to threat perception [9, 10].

Irritability is included in the negative valence system of Research Domain Criteria (RDoC) as “frustrative non-reward”. According to this neuroscience-based classification, irritability is an excessive response of an individual faced with the impossibility to achieve an expected goal. As such, it has been linked to dysregulations of the autonomic nervous system [11] and the reward network. Another pathophysiological line of research conceptualizes irritability as an aberrant response to threat (e.g., irritable individuals may attack more rapidly and in broader contexts) [12].

Despite the high prevalence and health issues related to irritability, there is little treatment research on this topic. Developing evidence-based non-pharmacological treatment options for children suffering from severe, chronic irritability is therefore a particularly important target for clinical research. One of the first steps in achieving this goal includes testing new therapies against extant interventions. Parent training programs decrease aggressive behaviors in children [13] and are therefore good candidates for the improvement of irritability. Low parental warmth, lack of positive parental emotion socialization, and high parental expressed emotions have been linked with irritability in children [14, 15] and could therefore be targeted in parental programs.

Research on the biological and psychological mechanisms mediating irritability is also needed to further improve the specificity of therapeutic actions [16].

### Parent management training (PMT)

Parent Management Training (PMT) is the gold-standard treatment for disruptive behavior disorders in children and adolescents. Barkley’s program is a PMT specifically designed for parents of defiant children [17, 18]. It can be performed either in a group (involving 6–8 families) or on an individual basis (one family). Generally, the program consists of 10–12 bi-monthly, 90-min training sessions. The objective is to train parents to cope with the child’s behavioural difficulties by teaching them effective control strategies that are coherent and adapted to the “deviant” behavior of their children; this reduces the intensity and the repercussions of undesirable behavioural events within the family. Numerous studies have demonstrated the efficacy of Barkley’s program. Following group training, parents report fewer behavior problems and with lower intensity [19]. The environment surrounding children with behavioural or emotional disorders must be as structured as possible. Disorganized, chaotic, and unpredictable environments can aggravate symptoms and favor the development of comorbidities. Hence, the importance of starting care via parental training can assure a good adjustment between the family environment and the special needs of the patient [20]. A Cochrane review involving 284 children (ages 5 to 18) found that parental guidance programs seemed to improve child behavior and reduce parental stress [13].

### Non-violent resistance (NVR)

The NVR psychological therapeutic model was developed by Pr. Haim Omer from the University of Tel Aviv to help parents cope with their child's violent and risky behaviors [21, 22]. Along with increasing parental presence and adherence to clear rules, the program’s core features are parental self-control and emotional support. In the NVR program, parents recruit supporters to help them dealing with their children’s problematic behaviors. Developing emotional control means the parent opposes non-violent resistance to the child's behavior, without escalating into an explosive situation. Several studies have demonstrated the efficacy of NVR across a variety of social and cultural settings (for review see [23]). NVR was shown to be effective at reducing violence and other externalizing symptoms, as well as parent–child escalation and parental helplessness. NVR treatment also helped parents to increase their positive attitudes toward the child, even in situations where parent–child interactions had become unattainable because of chronic conflict. The intervention showed high levels of feasibility and acceptability, and parents were very satisfied. Related to NVR is the concept of vigilant care [24]. This concept was developed in response to criticisms of parental monitoring, which, in some cases, has been found to lead to negative consequences such as increased parent–child conflict and excessive parental control. Vigilant care emphasizes the gradations of parental vigilance, from open attention through focused attention to active protection. In this way, parents move between different levels of involvement in response to danger signals detected from their child. Targeting parents whose children show severely disruptive behavior in the home setting [25], we aim to test both NVR and vigilant care, and to compare them to standard PMT and treatment as usual (TAU).

### Rationale

Based on the above literature, we conclude that parental skills training with PMT (which typically occurs via Barkley program in France) demonstrated various degrees of success in the treatment of disruptive behavior and emotional disorders. However, specific studies examining irritability as the main efficacy endpoint are needed.

The NVR program has specific features of particular interest for children and adolescents with severe irritability. In particular, the program outcomes include parental non-violent resistance and emotional regulation in responding to the child’s behavior. That is, the specific communication skills and techniques trained with the parents are usable in situations where the child or adolescent is involved in severe disruptive behavior and emotional distress.

Our team has developed a group format of the parent program based on Non-Violent Resistance and Vigilant Care through a collaboration with Professor Haim Omer. This NVR program has already been used in children with predominantly intra-familial ODD/CD in pilot clinical groups and in research settings and has shown excellent acceptability and positive feedback from participants.

Our goal is to rigorously test the NVR program in a group-format and against the most common comparator therapy in France, Barkley parental skills training program (PMT), and treatment as usual (TAU). We hypothesize that both the NVR and PMT programs will show therapeutic benefits on irritability in comparison with TAU. As this project will take place in the context of SARS-CoV2 pandemic, the parent programs will be held in the form of 2-day webinars with rehearsal sessions. We also expect that this online format will meet the expectancies of many parents because of its accessibility. This study, called RESist against Irritability Superiority Trial (RESIST RCT) will also examine other clinically relevant endpoints, as well as safety and acceptability of both the NVR parent group-format program and the standard PMT; thus, it will provide additional data for the clinical potential relevance and implementation of these programs.

### Explanation for the choice of comparators {6b}

In this trial, two presumably active groups are compared, the PMT group and the NVR group, against TAU. Parents in the TAU group will be offered to participate in PMT or NVR training in their respective inclusion centre after completion of the last study assessment.

### Objectives {7}

The main objective of this study is to assess the impact of non-violent parenting resistance training (NVR) and standard parenting management training (PMT) versus “usual treatment” (TAU) in improving irritability in children and adolescents with a baseline parent-rated affective reactivity index (P-ARI) greater than or equal to 4 in the context of ADHD, ODD, CD, mood / anxiety disorders or DMDD at 3 months after the program completion.

Main study hypothesis:

Both NVR and PMT are superior to TAU in improving irritability between baseline and 3 months after the program completion.

The secondary objectives of this study are:

- Assess improvement of irritability at different time-points (e.g., post completion).
- Assess improvement of irritability according to different informants (e.g., parents, child).
- Assess improvement of parental strategies.
- Explore putative mediators of therapeutic effect (e.g., parental expressed emotion, co-occurring disorders, personality).
- Assess acceptability of the interventions.

### Trial design {8}

**Fig. 1:**
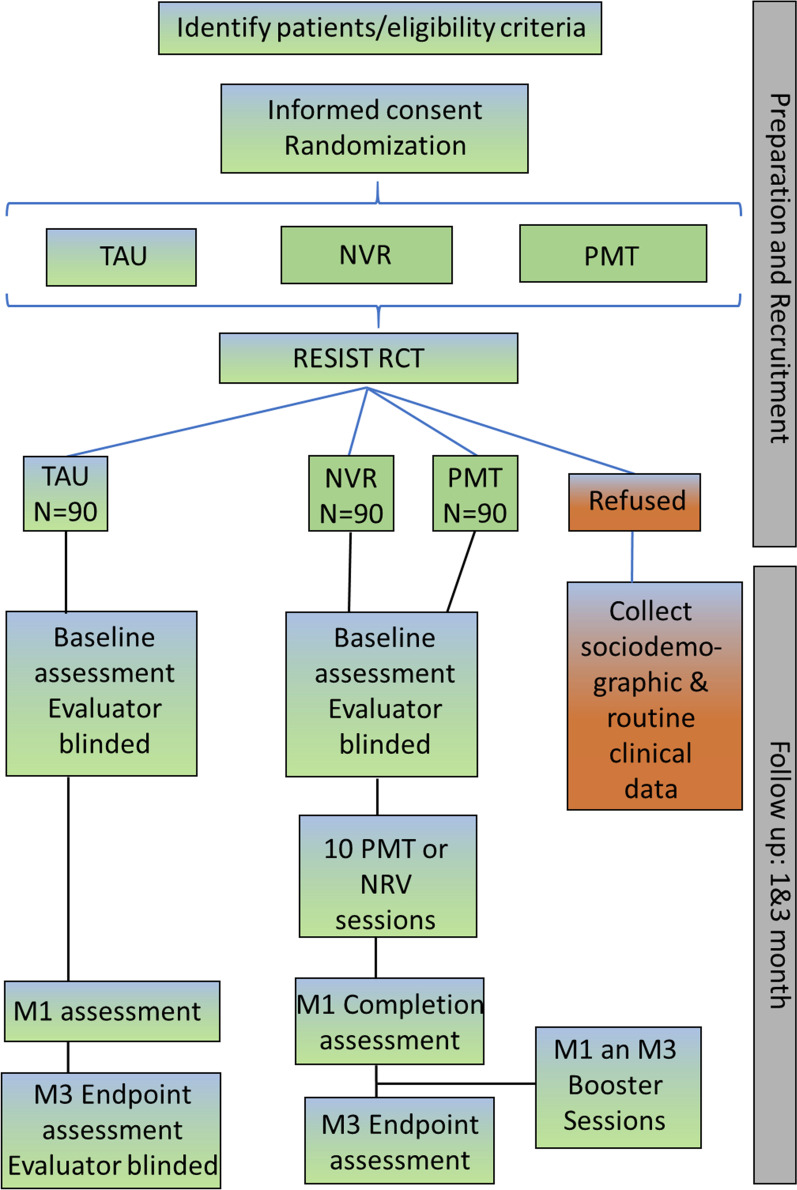
Flowchart. Flowchart of the RESIST RCT protocol testing the impact of non-violent parenting resistance training (NVR) and standard parenting management training (PMT) versus “usual treatment” (TAU) in improving irritability in children and adolescents with a baseline parent-rated affective reactivity index (P-ARI) greater than or equal to 4 in the context of ADHD, ODD, CD, mood / anxiety disorders or DMDD at 3 months after the program completion. N = planned/estimated sample size; M1 = at 1 month; M3 = at 3 months

The study described in this protocol is a prospective, multicentre, randomized (1:1:1), controlled superiority trial with evaluator blinding study conducted in France (Fig. 1).

## Methods

### Participants, interventions and outcomes

#### Study setting {9}

Patients are recruited at the child psychiatry units of the academic hospitals listed below. Study launching meetings are organized at each recruitment centre. Information (mailings, flyers, and posters) is distributed to partners of the different centres, including support groups, social services, and medico-social and associative structures. Clinicians of the participating centres are asked to screen their active files for eligible participants and to inform eligible parents and children about the RESIST RCT protocol. The study will then be presented to patients and their parents, along with appropriate information letters by the investigation team at the centre of Montpellier. If the patients/parents show interest in the study and give their consent a pre-inclusion visit is scheduled.

#### Eligibility criteria {10}

Inclusion criteria:

A subject has to meet the following criteria:

1. Male and female subject between 6 and 15 years old.
2. A confirmed K-SADS DSM-5 diagnosis of ADHD, ODD, CD, mood/anxiety disorder or DMDD. The request of a concomitant mental disorder allows to restrict this intervention to a clinical population.
3. A parent-rated ARI total score of 4 or above at baseline
4. A Clinical Global Impression-Severity score (CGI-S) of 4 or above (= at least moderately severe)
5. Persistence of irritability symptoms 6 month or above at baseline (this avoids including children with transitory irritability)
6. Stable treatment regimen (pharmacological and non-pharmacological) for 2 weeks prior to inclusion and during the trial

Exclusion criteria:

A subject will not be eligible for inclusion in this study if any of the following criteria apply:

1. Unavailability of parents or legal representative during the study period
2. Subjects with a DSM-5 diagnosis (clinical presentation or history) that is consistent with Schizophrenia or psychotic disorders or acute manic episodes.
3. Diagnosis of Autism Spectrum Disorder (ASD) in patients who are non-verbal and with IQ lower than 70
4. Known or estimated IQ < 70 or clinical diagnosis of intellectual disability
5. Subjects with severe irritability that are better accounted for by another factor, e.g., general medical condition(s) or direct effect of a substance (i.e., medication, illicit drug use), as determined by the clinical judgment of the investigator, or related to child abuse and/or neglect
6. Absence of informed consent form signed by at least one of the parents or legal representatives, and oral consent of the child
7. Inability to speak and comprehend French
8. Deemed unable to comply with the trial protocol
9. Participation in a structured parent program during the last 6 months

### Interventions {11a}

#### Standard PMT program

Our standard PMT program includes 10 online sessions and 2 booster online sessions, at 1 month and 3 months after completion. Sessions take place over two consecutive days.

Strategies for PMT group management are described in the manual book for clinicians:

Russel Barkley “Defiant children”, Guildford Press.

The session contents of the PMT program have been adapted to suit irritability and problematic behaviors and are described in the following section:

- First Session: Introduction about defiant disorders and irritability and presentation of participants (roundtable).
- Second Session: Psychoeducation about disorders associated with irritability including ADHD (Attention Deficit Hyperactivity Disorder), Oppositional Defiant Disorder (ODD), Conduct Disorder (CD), anxiety disorders, Autism Spectrum Disorders, Obsessive Compulsive Disorders, and Mood Disorders.
- Third session: Introduction to behavioural and cognitive therapy. Definition of operant conditioning and its impact in learning processes, including an explanation about the necessity of positive reinforcement of correct behavior.
- Fourth Session: Implementing a special time with the child, about 20 min daily, to get positive dynamics and share pleasant time.
- Fifth session: Learn to give effective orders, specifically adapting the way orders are given in order to increase the child’s compliance, e.g., avoid distractions, make orders as clear as possible, one order by time.
- Sixth session: Positive reinforcement of adaptive behavior. Parents learn to encourage their children when they display prosocial and adaptive behaviors in order to improve child’s autonomy, e.g., congratulating the irritable child as often as possible when he plays quietly or engages in cooperative attitudes.
- Seventh session: Set up a “point system” to increase the positive reinforcement’s impact and improve child’s motivation. Parents define positive behaviors that will be rewarded with special points. Accumulation of points may be exchanged against special non-material rewards.
- Eighth Session: Introduction to time-out periods. This strategy aims to face anger and temper outbursts in children.
- Ninth Session: Management of screens. Here we give strategies to set up a contract between parent and child to reinforce a controlled use of screens.
- Tenth Session: Management of schoolwork.

For the last five sessions, parents are separated into two groups: parents of adolescents and parents of younger children. The strategies used follow the same principles but are adapted to suit the age of the children (e.g., token reinforcement replaced by age-appropriate rewards).

Booster Sessions: review of strategies.

These sessions aim to review all strategies with parents, e.g., what was working or not, what limitations they had encountered, and what steps they could not overcome. Interactions between parents are very important at this stage because sharing their experiences is helpful for the entire group. Additionally, we can re-explain methods or strategies. We note that for many parents, time is necessary to understand the dynamics of parent management techniques.

#### NVR program

Our NVR program includes 10 online sessions and 2 booster online sessions (1 month and 3 months after completion). Sessions take place over two consecutive days.

The strategies for NVR group management are described in the manual book for clinicians:

N Franc, H Omer “Accompagner les parents d’enfants tyranniques, un programme en 13 séances» Ed Dunod 2013.

The NVR Program differs to the standard PMT such that therapists never emphasize the behavior or reaction of the child but always the reaction of parents. During each session, the principles of Non-Violent Resistance are rehearsed: “Your child wants to prove to you that you cannot control him, but you have to show that you can control yourself, and that your behaviour does not depend on his behaviour. Your success is to control yourself and to use non-violent strategies, this is your only goal. The objective is to receive kindness from your child and not to change his / her behaviour. Of course, this is what you deeply wish, but if this may happen, it will be later … first you have to change and to persist”.

This is a crucial point, as therapists have to change the paradigm of objectives. It means for example, that when a parent is desperate because they have tried using a new strategy (like a sit-in) and must cope with a temper outburst more violent than ever, we consider the situation as a very positive occurrence, as long as parent managed to keep a non-violent attitude.

Every session is structured as follows. At the beginning, parents are invited to give feedback about what happened at home with their child during the last week, and what strategy they could use. Therapists reinforce parents to consider why they are able or not able to apply strategies. At the end, therapists present and explain a new strategy to use at home: most of the sessions are practical (e.g., write a declaration, to set up a sit-in) and some are more academic (e.g., describe the principles of NVR). Slides are used in order to clarify and to assist attention. After academic presentation, we ask to parents to react, e.g., “do you agree with our observations? Do you recognize the dynamic functioning of your family? Do you think it will be possible for you to use this strategy? What would limit you? What solutions could other parents find in this situation?” Often, when a parent feels trapped and overwhelmed, we ask for input from other parents in finding effective strategies.

First Session: Introduction.

This session is a presentation of the program, introducing what kind of violent or deviant behaviors children and teenagers may present at home and in the community. We explain that severe irritability includes temper outbursts and how it can impact family life and parental confidence. We present other types of violence that may be associated with severe irritability such as verbal violence to parents or others; physical violence; and psychological violence such as suicide threats, playing with parental guilt, etc. We let parents share their common feelings of shame, guilt, lack of parental confidence, and self-depreciation. At the end of the session, a roundtable is organized. This session may be very emotionally intense when parents talk about their child to strangers.

Second Session: Psychoeducation.

Here we explain how irritability can be determined in child development and the involvement of temperamental background and genetic factors. This explanation is also necessary for parents to understand that their child is not angry “after them” but that he/she has difficulties in regulation of emotions that impacts them first, as they are the closest caregivers.

We also include psychoeducation on disorders associated with irritability, e.g., ADHD (Attention Deficit Hyperactivity Disorder), Oppositional Defiant Disorder (ODD), Conduct Disorder (CD), anxiety disorders, Autism Spectrum Disorders, Obsessive Compulsive Disorders, and Mood Disorders.

Third Session: Introduction to Non-Violent Resistance.

Here we explain the historical and sociological background of NVR and its application in psychotherapy. We give an academic bases about the work of Professor Haim Omer who has developed specific programs to cope with violence in children and teenagers at home and in the community. This session also includes explanations about the New Authority approach and the role of social support. We present schematics of violence escalation and ask them to describe the process of violence and authority in their family. Most parents report scenes of escalated violence to try to face temper outbursts of their child, and how they felt guilty and incompetent after such experiences.

We teach NVR principles, e.g., “your enemy is violence, not your child”, “you must not win, but you must persist”, “you have to strike while the iron is cold.” We explain that their reactions have to be postponed and never impulsive. An educative response is much more efficient when it is postponed, and this is a way for parents to show to their child that they are not dependent on their own emotions and they are capable of controlling themselves.

Fourth session: Writing and reading a declaration of non-violence.

This session relates to the implementation of a change in parental attitudes. Here, parents must write a declaration to their child stating that they will no longer accept violence. They can give examples of two or three unacceptable behaviors of their child. This is not judging but only descriptive. For example, “We don’t accept that you hit your little brother when you are angry.” By the same way parents must also engage that they will no longer use violence against their child. They inform that they will do their best to find solutions, that they will not stay alone in this situation, and that they will ask to other people to help because this is the role of parenting.

Parents are asked to write a declaration for the next session, to read it to the child; if they do not feel ready, they can read it to the group before reading it to the child. We prepare them to potential reactions, but we insist on the fact that reaction of the child (positive or negative) is not our objective.

Fifth session: Finding social support and creating a support-group.

To increase their level of non-violent authority, parents must engage other people (e.g., friends, colleagues, family, other parents, teachers) that may constitute a support group. This forces parents to talk about their child’s behavior with someone, which can be a difficult step due to shame and guilt. However, parents experience that they can get support and people involved in a support group, which can reinforce their parental confidence. The objective of a support group is to talk with the child as often as violent behaviors happen. It means for the child that the behavior is not secret, that the parents are supported, and there is a social judgement regarding their behavior. A support group may also be called in case of violence or temper outbursts to assist the parents at home.

Sixth Session: Management of temper outbursts.

Here we provide an introduction to emotion regulation in children and adults, and how this regulation may be disturbed, e.g., how the child tries to cope with his own emotions and why it may explode. We describe processes of temper outbursts and why parents should not interact with their child during crisis. We give them strategies to face the crisis with the priority of protecting themselves. We talk about the involvement of a support group either by phone or in-person when it is possible (neighbors). It is most important to avoid escalations in violence, to search for help, and to call to emergency services if it becomes dangerous for the child or family. After this session, if they need, we propose they write an “emergency letter” for parents so that emergency workers (police, fireman, etc.) can get information about the situation for when they arrive at the home.

Seventh session: Sit-In.

A “sit-in” is a way to react “when iron is cold”; it means to react after a temper outburst or violent behaviors, when situation has calmed. Parents need to experience that they can still be in an authoritarian position even if they do not react immediately after a violent behavior. They just need to say to the child when the problem happens that they do not accept it and that they will think about consequences and come back later. Then they will have a couple of days of delay to come into the room of their child, sit, and say “We are here because we do not accept what happened last time (e.g., when you hit your brother) and we want you to find a solution.” Then they have to stay calm and silent for 30 min. They are instructed to leave if they do not feel safe (and come back another day with a member of the support group). This action shows their new position of authority and their determination. Parents sometimes feel afraid so they engage in role-playing during the session.

Eighth Session: Taking care of yourself.

When parents need to cope with irritability and temper outbursts of their child, it often takes a big place in their personal life; they always try to anticipate what could make their child angry, they try to avoid actions or words, they have the feeling of “walking on eggshells”. Finally, they also have a bad perception of themselves as parents and sometimes as people. They have the feeling that they have to do more for the child and family, and less for themselves. This may create conditions of anxiety and depression disorders, that may decrease their authority and their emotion regulation. That is why we insist on the necessity of taking care of themselves, e.g., do things they like, leave their home few hours or days, etc. These are actions of resistance. We also insist on the need to get medical help for themselves if they have symptoms of depression or sleep disorders. In this session we have a special time (about 30 min) for an introduction to mindfulness.

Ninth session: Increasing parental presence.

Most of the time, parents try to avoid their irritable child because their time together is unpleasant. The child may complain, show signs of anger and sadness, and try to transform family time into conflicts. We explain to parents that presence is now the key to authority, i.e., they must spend time with their angry child and show interest in their activities and friends. When a child/teenager is defiant and does not respect family rules, we ask parents to use a new strategy to increase their presence. For example, if the teenager breaks curfew, parents should contact as many friends as possible to give a “parental presence message” instead of trying to contact their child on their mobile phone (who will usually not answer). In this way, parents show the child that they are present. If their child is angry, they can simply say “it is my duty of mother to try to know where you are”. They increase their level of authority and this may convince their child to respect family rules in the future.

For the last five sessions, parents are separated into two groups: parents of adolescents and parents of younger children. The strategies used follow the same principles but are adapted to suit the age of the children (e.g., token reinforcement replaced by age-appropriate rewards).

Tenth Session: Management of screens.

This is a challenge for parents with irritable children because the interruption of screen activity may create a temper outburst. We discuss with parents’ different activities their child may engage in on the Internet. Parents need to manage the screen time with contracts, otherwise they can contact their support group to find solutions; when a contract is not respected, a 24-h total privation of screen time is recommended. When a child’s behavior is too violent, the help of the support group is requested.

Booster Sessions: review of strategies.

These sessions aim to review all strategies with parents, e.g., working or problematic strategies, limitations they had met, and steps they could not overcome. Sharing different experiences between parents is helpful for the entire group at this stage. For many parents, booster sessions can reveal that time is a necessary component to understand the dynamics of non-violence.

### Treatment as usual (TAU)

The TAU group receives non-pharmacological and pharmacological therapies as usually provided in the participating centres. The TAU group participates in the assessments as described in the procedures.

### Criteria for discontinuing or modifying the allocated interventions {11b}

Subjects may be withdrawn from the study at the discretion of the investigator or sponsor due to safety concerns. In addition, a subject must be withdrawn if one of the following applies:

- Subject chooses to withdraw from the study at any time.
- Major violation of the study protocol.
- Other circumstances that would endanger the health of the subject if he/she were to continue his/her participation in the trial.

Reasons for withdrawals and discontinuation of any subject from the protocol must be recorded.

The evaluation of protocol violations and patient removal will be produced only at the blinded data review stage.

Main major violations are:

- Participant did not complete a minimum of 50% of the program’s modules, including the follow-up visits.
- Participant did not complete the main study assessments.

### Strategies to improve adherence to interventions {11c}

To improve adherence to the intervention protocols, routine visits will be made by monitors designated by the sponsor in order to check compliance with the protocol; the completeness, accuracy, and consistency of the data and adherence to Good Clinical Practice (GCP) requirements.

The principal investigator must ensure that electronic case report forms (e-CRFs) are completed in a timely manner and must allow periodical access to e-CRFs, patient records, drug logs, and all other study-related documents and materials.

The investigator will agree to provide laboratory assessment reports and results, and direct access to the subjects’ source data, which may exist in the form of hospital records, patient files, and notes.

### Relevant concomitant care permitted or prohibited during the trial {11d}

Throughout the duration of the study, the treatment regimen (medication or non-pharmacological therapies) will be kept stable whenever possible. In the case of a change, it will be traced in the e-CRF. Moreover, it is recommended that parents do not participate in other parental management training programs.

### Outcomes {12}

#### Primary endpoint

Change from baseline at 3 months of the Clinician-rated Affective Reactivity Index total score (CL-ARI) [26].

#### Secondary endpoints

Secondary efficacy endpoint at completion:

- Change from baseline to follow-up at 1 month of the program of the CL-ARI [26].

Secondary efficacy endpoints at 1 month and at 3 months follow-up:

- Change from baseline of the Clinical Global Impression Improvement (CGI-I) scale [27].
- Change from baseline in the Parenting and Familial Adjustment Scale (PAFAS) [28].
- Change from baseline of Parent-rated ARI (P-ARI) [29, 30] and Child-rated Cranky thermometers [31].

Exploratory efficacy endpoints and putative moderators and mediators:

- Initial score and change from baseline of specific Child Behavior Checklist (CBCL 6–18) (parent-rated) [32]: CBCL-internalizing, externalizing, and DP/Dysregulation short scale at 1 month (M1) and at 3 months (M3).
- Initial score of the personality dimensions assessed by parents (Hierarchical Personality Inventory for Children; HiPIC) [33]).
- Initial score and change from baseline of the parental expressed emotions and reflexivity rated with the Five-minute Speech Sample (FMSS) [34] at M3.
- Initial score and change from baseline in the Inventory of Callous Unemotional traits (ICU) [35].

Secondary acceptability endpoints:

- Observance.
- Acceptability (questionnaire for all parents and qualitative interview in a subsample of the ancillary RESIST-QUAL; the qualitative interview will be carried out after completion of the treatment).
- Discontinuation from the trial due to any cause and due to specific causes.

Other assessments:

- Socio-demographic and early development questionnaire.
- Medical history.
- Concomitant medications.
- K-SADS.
- Clinical Global Impression Severity (CGI-S) scale at baseline.

#### Pre-inclusion visit

The pre-inclusion visit can be done following a routine appointment in the centres or after a previous e-mail contact. The site investigator or clinician will give the family a first information about the research and hand out patient information and consent forms. An investigator from Montpellier will join the families by phone or videocall to answer further questions and check inclusion and exclusion criteria.

Informed consent and assent will be collected from parents, children and adolescents, before starting any trial-specific procedure through an online consent signature. Participants will be advised that research is entirely voluntary and that they can withdraw their participation at any time.

The centre will try to obtain consent from both parents. In case of consent from one parent only, this will be justified by the centre. The participation in the study remains possible for one parent.

At pre-inclusion, children are assessed to confirm their diagnostic status. This evaluation includes the Kiddie-Schedule for Affective Disorders and Schizophrenia (K-SADS) interview in the case it was not done in the 6 months prior to the pre-inclusion. The K-SADS is a semi-structured diagnostic interview designed to assess current and past episodes of psychopathology in children and adolescents according to DSM-5 criteria [36]. A french version has been finalized for DSM-5 diagnoses (download available at https://sfpeada.fr/k-sads-pl-dsm-5-version-francaise-2018/). Interviews will be carried out on site or online by trained master-level or doctorate-level clinicians.

At screening and during enrolment of the RESIST RCT, participants will be asked to maintain their current mental health treatment regimen for 4 weeks prior to the start of the program and through the second endpoint visit (e.g., second follow-up visit).

#### Baseline assessment: inclusion visit

After the signature of informed consent and verification of inclusion and exclusion criteria, the following baseline assessments will be completed by children and adolescents, parents, and clinicians by video-conference with the evaluator.

Parents’ assessments.

- Socio-demographic and early development questionnaire.
- Current medication status of the child.
- Hierarchical Personality Inventory for Children (HiPIC) [33].
- Parent-rated Affective Reactivity Index (P-ARI) [29].
- CBCL [32].
- Parental and Familial Adjustment Scales (PAFAS) [28].
- A short speech recording for the parental Expressed Emotion (EE) and reflexivity will be rated using the FMSS (putative mediator of treatment effect) [34].
- ICU [35].

Children’s/adolescents’ assessments.

- Cranky thermometers [31] (self-rated irritability with visual analogous scales).

Clinician’s assessments.

- Clinician-rated ARI (CL-ARI) [2].
- CGI-S [3].

#### Treatment groups

PMT and NVR online parent program sessions will be held by experienced clinicians from the University Hospital Centre (CHU) of Montpellier and will be open to clinicians from the recruitment sites for training purposes. The treatment programs are adapted from Barkley’s program for defiant children and teens [17, 37] and from the NRV program by N. Franc and H. Omer, both of which are available as manuals [25].

The group sessions of each program are coordinated by two clinicians. Each session will include an open question period facilitated by the presence of a real time chat.

Format of the group therapy: 10 online sessions of either NVR of PMT with up to 60 participants over two consecutive days (5 sessions per day) with additional booster sessions at 1 and 3 months after the 10th session.

Both treatment groups will be led by a tandem of experienced clinicians (psychiatrists/psychologists/nurses/occupational therapists) from the coordinating centre at Child and Adolescent Psychological Medicine (MPEA1) of CHU Montpellier. One clinician leads the session and the oral discussions, while the other clinician monitors the chat and participates in guiding the discussion.

The NVR parent group format program consists of 10 sessions and 2 booster sessions designed to develop a positive form of authority based on parental presence, a parental support network, and strategies of non-violent responses in order to avoid escalation while promoting reconciliation gestures.

The PMT program consists of 10 sessions and 2 booster sessions. PMT is an evidence-based treatment for disruptive behavior disorders in which the child’s social environment is modified according to principles of operant conditioning and contingency management. The parental response to the child’s behavior increases or decreases the likelihood of targeted behavior.

#### Follow-up assessments

Investigators involved in assessments will be blinded as regards program/treatment status. A full assessment in parents and children will be completed at a 3 months follow-up.

At 1 month the following assessments are scheduled:

Parents’ assessments.

- P-ARI [29].
- CBCL [32].
- PAFAS [28].

Clinician’s assessments.

- CL-ARI [26].
- CGI-I [27].
- Observance of treatment sessions will be recorded by therapists involved in each treatment group.

Children’s/adolescents’ assessments.

- Cranky thermometers [31] (self-rated irritability with visual analogous scales).

At 3 months follow-up:

Parents’ assessments.

- P-ARI; specific irritability severity [29].
- CBCL [32].
- Parental EE and reflexivity will be rated using the FMSS (putative mediators of treatment effect) [34].
- HiPIC [33].
- PAFAS [28].
- ICU [35].
- Current medication status of the child.

Childrens’/adolescents’ assessments.

- Cranky thermometers [31] (self-rated irritability with visual analogous scales).

Clinician’s assessments.

- CL-ARI [26].
- CGI-I [27].

### Participant timeline {13}

The participation timeline of the RESIST protocol is represented in Fig. 1.

### Sample size {14}

According to the results of Maire [36] and Stringaris et al. [29], the mean score of ARI was 4.31 in an ADHD population with a standard deviation (SD) of 2.72, mean of 7.2 (SD 1.0) in severe irritability, mean of 5.1 (SD 1.5) in bipolar disorder, and mean of 0.5 (SD 0.2) in normal controls. In a recent study, a clinical sample had a mean of 3.5 (SD 1.4) and children with a diagnosis of disruptive mood dysregulation disorder had a mean of 4.9 (SD 1.4) [2]. In a clinical trial of online PMT, the mean ARI-irritability score decreased from 7.2 (SD 2.6) to 3.75 (SD 2.1) [42].

The mean expected improvement in each active group is −2 units. Among the control group (treatment as usual, TAU), the expected variation is −0.5. According to the One-Way analysis of variance (ANOVA) model in Nquery software, taking a difference between-group of 1.5 (Variance of means V = 0.5) with a SD of 3, a total sample size of 77 subjects in each group will permit detection of a difference between groups with a global two-sided alpha = 0.05 and with a power of 90%. Considering a dropout rate around 15%, it will be necessary to include 270 subjects (90 subjects/group).

### Recruitment {15}

Study launching meetings and press releases will be organized at each recruitment centre. Information (mailings, flyers, and posters) will be distributed to partners of the different centres, including support groups, social services, and medico-social and associative structures. Clinicians of the participating centres will screen their active files for eligible participants in order to inform parents and children about the RESIST RCT protocol. The study will be presented to patients and their parents with appropriate information letters during a routine visit or via e-mail using patients’ contact information. If the patients/parents show interest in the study, a pre-inclusion visit is scheduled.

## Methods

### Assignment of interventions (for controlled trials)

#### Allocation

##### Sequence generation {16a}

The randomization will be done with Ennov-Clinical software (CS-random module). The randomization will be centralized, available online, and stratified by minimization based on centre and on two age classes (6–11 and 12–15 years) and medication status (according to major classes of psychotropic), balanced with a 1:1:1 ratio. Each subject (i.e., here duo or trio of child and parents), who meets the eligibility criteria and signed informed consent, will be randomly assigned to one of the three study groups by the study investigator.

##### Concealment mechanism {16b}

The allocation sequence will be implemented after completion and signature of the baseline visit on the e-CRF.

##### Implementation {16c}

The randomization will be done by the clinical research centre of Montpellier University Hospital after confirmation of patients’ and parents’ participation.

Participants will be enrolled by the centre of Montpellier during the pre-inclusion. This visit can be done following a routine appointment in the centres or after a previous e-mail contact. The site investigator or clinician will first give the family information about the research and provide patients with information and consent forms. An investigator from Montpellier will join the families by phone or videocall to answer further questions and check inclusion and exclusion criteria.

Informed consent and assent will be obtained from parents, children, and adolescents before any trial-specific procedures are performed. Participants will be advised that research is entirely voluntary.

The centre will aim to obtain consent from both parents, although the participation for one parent only is possible. In the latter situation, the centre will document it.

#### Blinding

##### {17a}

After the signature of informed consent and verification of inclusion and exclusion criteria, the investigators involved in the assessments will be blinded to the type of program received by the parents. This aspect will be monitored throughout the study and parents will be instructed before every assessment to avoid disclosing the type of therapy received. Investigators will be different from the therapists involved in NRV and PMT.

##### {17b}

There is no procedure for revealing a participant’s allocated intervention during the trial, because it is not a protocol targeting a pharmacological treatment. Therefore, it did not seem necessary to provide circumstances for and an unblinding protocol.

## Methods

### Data collection, management, and analysis

#### Data collection methods {18a}

Each assessment will be collected for each participant in accordance with the study data collection form by the investigator(s) or the clinical study technician(s) in charge of the study.

#### Socio-demographic assessments

The socio-demographic and early development questionnaire used for clinical and research purposes at the coordinating site at the CHU of Montpellier will be used.

### Questionnaires and Scales

#### Primary outcomes

##### Affective reactivity index (ARI)

The parent-rated ARI [29, 30] is a 6‐item scale that both clinicians and parent answer. The ARI asks about symptoms of irritability and includes a 7 item assessing impairment due to irritability. The scale shows excellent internal consistencies with Cronbach's alphas 0.89 (parent‐reported) and 0.90 (clinician-reported). The ARI has been translated into French by Maire [36].

The clinician-rated ARI [26] has been chosen as the main efficacy variable of the study to rate all available information while keeping main outcome variable (group allocation) blinded. The CL-ARI contains verbal prompts and examples to lead the administrator through the items. A total of 12 items query the frequency, duration, and severity of mild, moderate, and severe temper outbursts and irritable mood between outbursts, as well as functional impairment in the home, school, and peer settings.

##### Clinical global impression (CGI)

The CGI is a clinician-administered tool used to assess the symptoms of mental health disorders [27]. This tool provides a scoring of initial severity on the CGI-Severity Scale (CGI-S) from 1 to 7; subsequent improvements over time during treatment are rated using the CGI-I.

Scoring for the CGI-S is as follows: 1 = normal, not ill at all; 2 = borderline ill; 3 = mildly ill; 4 = moderately ill; 5 = markedly ill; 6 = severely ill; and 7 = among the most extremely ill patients. At subsequent study visits, clinicians use the CGI-I 7-point scale to rate the patients’ total improvement based on comparison with their baseline assessment from 1 = very much improved to 7 = very much worse.

##### Child behavior checklist (CBCL)

The CBCL is a standardized form that parents complete to describe their children’s behavioural and emotional problems [32, 38]. We will use the problem items for ages 4 to 18 years that can be completed in approximately 10 min. The data obtained with the CBCL are summarized on a profile that displays the parents’ ratings of each item. The profile also displays the child’s standing on syndromes of problems that were derived from statistical analyses of CBCLs in large numbers of clinically referred children. In order to capture changes, the CBCL is completed for the preceding month (instead of 6 months).

##### Parenting and familial adjustment scales (PAFAS)

The PAFAS [28] is a 36-item questionnaire designed to assess changes in parenting practices and parental adjustment in the evaluation of individual or group parenting interventions. The inventory consists of the parenting scale with two subscales measuring parenting practices and parent–child relationships and of the Family adjustment scale measuring parental emotional adjustment and partner support. A French version has been used in a study of ADHD and irritability [39].

##### Personality assessments (HiPIC and ICU)

To measure childhood personality, we include the Hierarchical Personality Inventory for Children (HiPIC) [33], an age-appropriate version of the Five-Factor Model (FFM) of personality. The HiPIC comprises of 144 items rated on 5-point Likert scales, assessing 18 facets hierarchically organized under five higher-order dimensions that are conceptually and empirically related to the adult FFM-domains: Emotional Stability (comprising the facets Anxiety and Self-confidence), Extraversion (Shyness, Optimism, Expressiveness, and Energy), Conscientiousness (Achievement Motivation, Concentration, Perseverance, and Orderliness), Benevolence (Egocentrism, Irritability, Compliance, Dominance, and Altruism), and Imagination (including Creativity, Curiosity, and Intellect).

The ICU [35] by parent report is a 24-item questionnaire evaluating callous and unemotional traits in children and adolescents, which is reported to change with parental programs and predict quality of treatment response. Respondents are asked to rate their agreement with the different items on a 4-point Likert Scale. The total score will be used in this study.

##### Cranky thermometers

The cranky thermometers are visual analogue scales measuring irritability in youth. The first scale, Cranky Now, measures current irritability, and the second, Cranky Two Weeks, measures peak irritability within the last two weeks. Positive associations have been found between cranky thermometers and irritability scores (as determined by Kiddie Schedule for Affective Disorders and Schizophrenia) and Affective Reactivity Index scores. Results suggest that the cranky thermometers are rapidly administered, have promising psychometric properties, and demonstrate utility in measuring irritability in clinical and community settings [31].

### Other assessments

#### Five-minute speech sample (FMSS)

Expressed emotion (EE) refers to the extent to which family members express critical/hostile and emotionally over-involved attitudes and remarks toward a specific person. It has been shown to be a predictor of patient relapse in those suffering from a range of mental disorders [40]. The 5-min speech sample requires a parent to speak for 5 min, without interruption, about their relationship and attitude toward the child. The underlying notion is that what the family member says and how he or she speaks about the target person will reveal the amount of expressed emotion typically present in the household. A coding system was developed to score facets of emotional expression such as criticism and emotional over-involvement [41].

##### {18b}

A subject may withdraw from the study at any time at their own request or at the discretion of the investigator for safety, behavioural, or administrative reasons. In the event that an included subject is withdrawn, the investigator should make every effort to perform a final study visit and complete the assessments and procedures outlined in the Table 1 for the follow-up visit.

**Table 1:** Participant timeline {13}

Visit	V0 Pre-inclusion	V1 Inclusion and randomisation	Program (NVT or PMT)	V2 M1	V3 M3 Follow-up
Day	D-90 to D0	D0	D1–D2	D30 ($\pm 15)$ = 1st booster session	V2 + 60 ($\pm 15)$ days = 2nd booster session
Protocol explanation	X				
Informed consent form		X*			
Inclusion/exclusion criteria		X*			
Demographic data		X			
Medication status		X		X	X
Parent ratings and assessments					
ARI-parent		X*		X*	X*
CBCL		X*		X*	X*
PAFAS		X*		X*	X*
HiPIC		X*			
5-minute speech sample (FMSS)		X*			X*
K-SADS	X				
ICU		X*			X*
Child ratings and assessments					
K-SADS	X				
Cranky thermometers		X*		X*	X*
Clinician ratings					
ARI – clinician		X*		X*	X*
CGI-I				X*	X*
CGI-S		X*			
Presence at sessions				X*	

Time and events of RESIST RCT for the assessment of primary and secondary endpoints; V0 = pre- inclusion visit; V1 = inclusion visit; V2 = booster session at 1 month (1 M); V3 = booster session at 3 months (3 M); D = day(s).

##### Data management {19}

Source documents are original documents and patient records from which patient data are reported in the e-CRF. The investigator must commit to allow direct access to data sources in the study during inspections, audits or inspections. For each participant there is an e-CRF, developed from the software Ennov Clinical to control data quality at entry. This software complies with the FDA recommendations on computerized systems for managing clinical trials ("Guidance for Computerized systems Used in Clinical Trials"), electronic signature ("21CFR part 11") and other domestic and international norms (CDISC, ICH, BPC 2001/20/CE…). The connection is via a username and password unique to each specific user and giving access only to the data of the centre’s user.

An audit trail function is included allowing a supervision and traceability of all actions from all users. The encrypted data is transmitted to the data-management centre via a secure internet connection. The e-CRF will be designed to capture all relevant medical information from patients included in the project.

The investigator must allow direct access to data sources in the study during inspections and audits.

The data manager will perform additional computerized consistency tests to detect the presence of non-standard, missing, aberrant, or incoherent data. These tests will be executed regularly during the participants’ recruitment and monitoring. Each identified incoherence will be the subject of a request for clarification by the researcher.

### Pseudonymization of data

In order to preserve anonymity, participants will be identified on the e-CRF by a unique identification number, the first letter of their first name, the first letter of their last name, their gender and their year of birth. A list of names of participants will be kept in the investigator's file. The investigating team will ensure that the anonymity of each participant in the study is guaranteed. The information will be collected for each participant in accordance with the study data collection form by the investigator(s) or the clinical study technician(s) in charge of the study.

### Data processing

Individual data needed for the study analysis must:

- be entered in the e-CRF and the non e-CRF as they are obtained, for both clinical and paraclinical data.
- be anonymized by the investigator.
- be authentified by an electronic signature of the investigator.
- all be entered, and missing data must be justified.

Data entered in the e-CRF are verified and validated by the Clinical Research Associate from source documents.

The Data Manager performs additional computerized tests to ensure completeness, consistency and reliability of data during the recruitment and follow-up of patients. All consistency tests are defined by the data manager in a validation test book and are approved by the investigator, the statistician, and the clinical research associate, before programming.

Each identified inconsistency will be included in the data clarification/resolution forms by the data-manager, posted online. The investigator receives an email informing him that requests for corrections are posted on the website of data entry. The investigator and / or clinical research associate connects to the website and confirms or corrects inconsistencies.

### Data coding

Data coding is integrated into e-CRF. Drugs are coded when entered using their international name (based on the WHO database). Some data are recoded by the study biostatistician before analysis.

### Data storage

Data will be saved by the Clinical Research and Epidemiology Unit (CREU) of Montpellier Hospital. Server location: Software and database reside on OVH servers (Dedicated cloud ISO 27001).

### Datatabase lock

Once quality assurance procedures have been completed, database lock will occur according to the procedures of the clinical research and epidemiology unit of Montpellier Hospital, and a database lock certificate is edited.

### Statistical methods

#### {20a}

A statistical analysis plan (SAP) will be prepared by the sponsor. Statistical analyses will be performed with SAS® version 9.4 or higher (SAS institute, North Carolina, USA).

Data will be analysed under the responsibility of the Unit of the Clinical research of CHU Montpellier with the SAS® software (SAS Institute, Cary, NC, USA).

The statistical analysis plan (SAP), covering all the analyses to be performed on all data, will be written before database lock.

Population sets

Four analysis, sets will be defined for this study:

1. The Enrolled Set (ES) will include all subjects who sign an Informed Consent form.
2. The Randomized Set (RS) will include all subjects who are randomized.
3. The Full Analysis Set (FAS) will include all subjects who are randomized and have a valid primary efficacy measurement.
4. The Per Protocol Set (PPS) will include all subjects who are in the FAS and who do not have an important protocol deviation that could affect the evaluation of the main outcome.

The FAS will be the primary set used for analyses/summaries of the primary efficacy variable, as well as for all secondary and other efficacy variables; the robustness of the analyses/summaries on the primary and key secondary efficacy variables will be assessed using the PPS.

The ES will be used for summarizing only the relative frequency of screen failures. The ES or RS will be used to establish the flow chart.

### Efficacy analyses

#### Primary endpoint

The full analysis set will be used for the primary endpoint.

The primary endpoint is the change from baseline (inclusion visit) at end of treatment (last visit) in the Clinician ARI total score. An analysis of variance will be performed to study the evolution of Clinician ARI total score (dependent variable) according to the three treatment groups and baseline value of ARI as a covariate. Each experimental group will be compared to the control group.

The treatment effect will be estimated by the least square mean (LSMean) of the difference with its two-sided 95% confidence interval. A graphical display of the LSMean and associated 95% confidence intervals for each treatment will be presented. In the event that marked deviations from the assumption of normality are observed, an additional analysis on the primary efficacy variable will be performed using non-parametric method.

#### Secondary endpoints

Secondary efficacy endpoints (change at completion of Clinician and Parent ARI, CGI-I score, PAFAS, change in specific CBCL 6–18, FMSS, and personality test scores) will be analyzed using the same methods as the primary endpoints.

### Exploratory analyses {20b}

#### Observance

The number of performed sessions will be described. The discontinuation from the trial due to any cause (or due to specific causes) will be also reported and compared between groups.

Analyses for additional subgroups are not planned in the main study.

#### Demographic and baseline characteristics {20c}

To ensure comparability, patient demographic and baseline characteristics (including medical history and prior medications) will be summarized by group using descriptive statistics. For continuous variables, descriptive statistics (*n*, mean, standard deviation, standard error, median, minimum, and maximum) will be provided. For categorical variables, patient counts and percentages will be provided. Categories for missing data will be presented if necessary.

In the case of non-comparability for one or more parameters, an adjustment will be made on this/these parameter(s) for analysis of the judgment criteria. A multivariate linear regression model may be performed to account for potential confounders.

The type of missing data for the variables of interest will be determined. Conditional rejection of the hypothesis NMAR (Not Missing at Random)—the assumption MCAR (Missing Completely at Random) against MAR (Missing at Random)—will be tested by studying the relationship between the variable status (missing or not) and the values of the covariates. If the missing data is type MAR or MCAR, a multiple imputation (Rubin, 1987) will be implemented for a sensitivity analysis. In order not to increase the database and to get a good estimate of the variance parameters, five imputations will be realized.

### METHODS: Oversight and monitoring {21a}

Data monitoring will be carried out by an independent safety monitor who will ensure the compliance with the trial regulatory aspects. Additionally, they will verify data collection. The safety monitor acts as a representative of the study promotor.

- Site training and monitoring procedures

The monitor must present the protocol and all procedures related to the study during an initial visit before the first patient is included. A CRF of completion guidelines will be provided to the Investigator.

- Direct access to source data/original documents

The monitor will be allowed to have access to all source documents needed to verify the entries on the e-CRF and other protocol-related documents.

- Accuracy and Reliability of Data

To ensure accurate, complete, and reliable data, the sponsor (or their representatives) will do the following:

- Provide instructional material to the study sites, as appropriate.
- Provide a start-up training session to instruct the investigator(s) and study coordinator(s). This session will provide instruction on the protocol, the prompt, and full completion of the clinical report forms, study procedures, and the transmission of data in a timely manner to the clinical database for statistical analyses.
- Make periodic visits to the study site.
- Be available for consultation and stay in contact with the study site personnel by mail, telephone, and/or fax.
- Review and evaluate Case Report Form data and use.
- Conduct quality review of database.

#### Interim analyses {21b}

Statistical analysis will be performed by the Clinical Research Unit of the CHU Montpellier, with the SAS and R software after the freezing of the database, the approval of the review data and the approval of the statistical analysis plan. Handling of modifications to initial SAP will be updated and finalized in the statistical analysis plan.

#### Harms {22}

Any adverse events/adverse reactions/incidents related to the usual care of the patient will be declared by a health professional to the various circuits of sanitary vigilance applicable to each product or practice concerned (vigilance of the care, pharmacovigilance, materiovigilance, hemovigilance, cosmetovigilance, etc.) in conformity with the regulations in force.

#### Auditing {23}

The purpose of an audit is to confirm that the study is conducted as per protocol, International Conference on Harmonisation-GCP and applicable regulatory requirements; that the well-being and the rights of the subjects enrolled have been protected; and that the data relevant for the evaluation of the investigational product have been recorded, processed, and reported in compliance with the planned arrangements. The investigators will permit direct access to all study documents, drug accountability records, medical records, and source data.

#### Ethics approval and consent to participate {24}

Ethical approval for the trial was granted by the “Comité de Protection des Personnes Sud-Ouest et Outre-Mer” (Board affiliation: CPP SUD-OUEST ET OUTRE6MER 1).

The site investigator or clinician will give the family a first information about the research and hand out patient information and consent forms. Informed consent and assent will be collected from parents, children and adolescents, before starting any trial-specific procedure through an online consent signature. Participants will be advised that research is entirely voluntary and that they can withdraw their participation at any time. The centre will try to obtain consent from both parents. In case of consent from one parent only, this will be justified by the centre.

#### Protocol amendments {25}

GCP requires that the clinical protocol, any protocol amendments, the informed consent, and all other forms of patient information related to the study (e.g., advertisements used to recruit patients) and any other necessary documents are reviewed by an Independent Ethics Committee (IEC)/Institutional Review Board (IRB). Any revisions/amendments to the protocol will not be permitted without prior approval by the study steering committee. Any amendments to the protocol will require Institutional Review Board/Independent Ethics Committee (IRB/IEC) approval prior to implementation of any changes made to the study design.

#### Consent or assent {26a}

Informed consent and assent are obtained from the parents and children, respectively, before starting any trial-specific procedure. All participants are advised that participation in research is entirely voluntary, and that they can withdraw their participation at any time. Families are not paid for their participation. The child’s psychiatrist or pediatrician initially presents the study. The protocol is then explained in detail by the clinical investigator before the signature of the informed consent by parents and child are collected using an online consent signature.

##### {26b}

N/A.

##### Confidentiality {27}

Individual subject medical information obtained as a result of this study is considered confidential and disclosure to third parties is prohibited, unless directed so by local regulations. Such medical information may be given only after approval of the subject to the subject’s physician or to other appropriate medical personnel responsible for the subject’s well-being. Exception to this are the Competent Authority; they have access to such medical information, as part of their inspection activities, without the consent of the subject.

The sponsor shall not disclose any confidential information on subjects obtained during the performance of their duties in the clinical study without justifiable reasons. The sponsor affirms the subject’s right to protection against invasion of privacy. Only a subject identification number and/or initials will identify subject data retrieved by the sponsor. However, the sponsor requires the Investigator to permit the sponsor, sponsor’s representatives, the IEC, or when necessary, representatives of the regulatory health authorities to review and/or to copy any medical records relevant to the study.

The sponsor will ensure that the use and disclosure of protected health information obtained during this clinical study complies with the legislation related to the privacy and protection of personal information.

##### Declaration of interests {28}

The authors have no financial or any type of competing interests to disclose.

##### Access to data {29}

The investigator must commit to allow direct access to data sources in the study during inspections, audits or inspections. An audit trail function is included allowing a supervision and traceability of all actions from all users. The investigator must allow direct access to data sources in the study during inspections and audits. To ensure confidentiality, data dispersed to project team members will be blinded of any identifying participant information. Project data sets will be housed by the Clinical Research Unit of Montpellier University.

##### Ancillary and post-trial care {30}

Participation in the clinical-trial does not interrupt previous care in the participating centres, assuring that post-trial care is guaranteed.

#### Dissemination plans {31a}

##### Study report

Data analysis will be done by study methodologist. A final report dated and signed by investigator will be sent to sponsor, which will send it to competent authority within 12 months after the end of study.

### Publication

The CHU of Montpellier is the data owner and any oral or written communication of research must receive the prior agreement of coordinator investigator and sponsor. The CHU of Montpellier must be referenced as research sponsor.

Publications must mandatory precise: "This study was supported by a grant from the French Ministry of Health (PHRC 2019-0150)". Template of writing of address of CHU of Montpellier in publications: CHU Montpellier, department /service, town, F-post code, country.

### Results communication

In accordance with the law n°2002–303 of the 4th of march 2002, participants are informed, at their request, of general results of research by the investigator.

For research purposes, the investigator can provide the full protocol, participant level-data, and statistical code on request.

#### Authorship eligibility guidelines and any intended use of professional writers {31b}

We will list all the professionals that have participated in the RESIST study.

- with substantial contributions to the conception or design of the work; or the acquisition, analysis, or interpretation of data for the work;
- Drafting the work or revising it critically for important intellectual content; AND
- Final approval of the version to be published; AND
- Agreement to be accountable for all aspects of the work in ensuring that questions related to the accuracy or integrity of any part of the work are appropriately investigated and resolved

We do not intend to hire any professional medical writers.

#### Plans to give access to the full protocol, participant level-data, and statistical code {31c}

In accordance with the law n°2002–303 of the 4th march 2002, participants are informed, at their request, of general results of research by the investigator.

For research purposes, the investigator can provide the full protocol, participant level-data, and statistical code on request.

## Discussion

This randomized controlled trial aims to investigate whether two intervention therapies, PMT and NVR, can improve irritability in children and adolescents with a baseline clinician Affective Reactivity Index 4 or higher in the context of ADHD and other emotional and behavioural disorders, compared to TAU.

Secondary, we will assess i) improvement of irritability at different times and according to different informants (parents, children, clinicians); ii) improvement of parental strategies; and iii) acceptability of the interventions, exploring possible mechanisms of the therapeutic effect.

Irritability in children and adolescents is associated with clinical severity and particularly poor outcomes [1, 5]. Chronic irritability was identified by both DSM and ICD taskforces as a topic of interest, making it a circumstance likely to foster specific research efforts [43]. Therefore, irritability represents a new treatment target and a clinically relevant trans-diagnostic dimension associated with significant personal, social, and family burden [44]. Parent management programs are a cornerstone of the evidence-based treatment of behavioural disorders and have also shown efficacy for internalized and externalized symptoms [13, 17–22, 25, 45]. Moreover, parents play an active role in both PMT and NRV, since those therapeutic approaches are built on parent empowerment [17, 21, 22, 25]. For this reason, we expect effects on parental attitudes and emotional expression. In fact, parent management programs are adaptable to increase emotional regulation strategies, but they are presently not easily available in France; NVR programs are even less implemented. The implementation of a group-delivered parental NVR program could represent an innovative option for children with ADHD, as it has mainly been used in an individual context. Already in different countries, the principles of NVR have been successfully implemented in diverse settings such as psychiatric wards and in classrooms to reduce dysregulated behavior and coercive escalation [46]. We believe that NRV will offer an alternative to the classical operant conditioning techniques used in PMT for the treatment of behavioural disorders [17].

The comparability of our results from the different centres embedded in the RESIST RCT project will be enhanced by the webinar format of intervention therapies; the format will be delivered by the same set of instructors permitting the simultaneous participation of parents from different sites.

By organizing training of both PMT and NRV across major university centres, we hope to raise interest and develop skills in mental health professionals to use evidence-based psychotherapeutic approaches for children suffering severe mental health conditions.

In summary, a direct benefit is expected in both treatment groups for patients, parents, caregivers, and for the staff trained in the programs.

## Data Availability

Not applicable.

## Abbreviations

ADHD: Attention-Deficit/Hyperactivity Disorder
AE: Adverse Event
ARI: Affective Reactivity Index
ASD: Autism Spectrum Disorder
CBCL: Child Behavior Checklist
CD: Conduct Disorder
CRT: Cluster Randomized Trial
CGI-I: Clinical Global Impression-Improvement
CGI-S: Clinical Global Impression-Severity
D: Day
DMDD: Disruptive Mood Dysregulation Disorder
DSM: Diagnostic and Statistical Manual of Mental Disorders
e-CRF: Electronic Case Report Form
FMSS: Five Minutes Speech Sample
GCP: Good Clinical Practice
HiPIC: Hierarchical Personality Inventory for Children
ICD-10: International Classification of Diseases
IRB/IEC: Institutional Review Board/Independent Ethics Committee
IQ: Intelligence Quotient
K-SADS: Kiddie-Schedule for Affective Disorders and Schizophrenia
LSLV: Last Subject’s Last Visit
mITT: Modified Intend to Treat
NVR: Non-Violent Resistance
ODD: Oppositional Defiant Disorder
PMT: Parent Management Treatment
PP: Per Protocol
PAFAS: Parental and Familial Adjustment Scales
RCT: Randomized Clinical Trial
RDoC: Research Domain Criteria
SAE: Serious Adverse Event
SAP: Statistical Analysis Plan
SD: Standard Deviation
TAU: Treatment-As-Usual
